# Supplementary material for: Lethal microbial blooms delayed freshwater ecosystem recovery following the end-Permian extinction
Source: Nat Commun. 2021 Sep 17;12:5511. doi: 10.1038/s41467-021-25711-3 (PMC8448769; doi:10.1038/s41467-021-25711-3)
Supplement: Supplementary file 1 — Supplementary Information [file 41467_2021_25711_MOESM1_ESM.pdf]

Supplementary Information

Lethal microbial blooms delayed freshwater ecosystem recovery following the end-Permian extinction

Chris Mays[1], Stephen McLoughlin[1], Tracy D. Frank[2], Christopher R. Fielding[2], Sam M. Slater[1], Vivi Vajda[1]

[1] Department of Palaeobiology, Swedish Museum of Natural History, Box 50007, SE-104 05 Stockholm, Sweden.
[2] Department of Earth & Atmospheric Sciences, University of Nebraska-Lincoln, 126 Bessey Hall, Lincoln, NE 68588-0340, USA.

Correspondence and requests for materials should be addressed to C.M. (email: chris.mays@nrm.se).

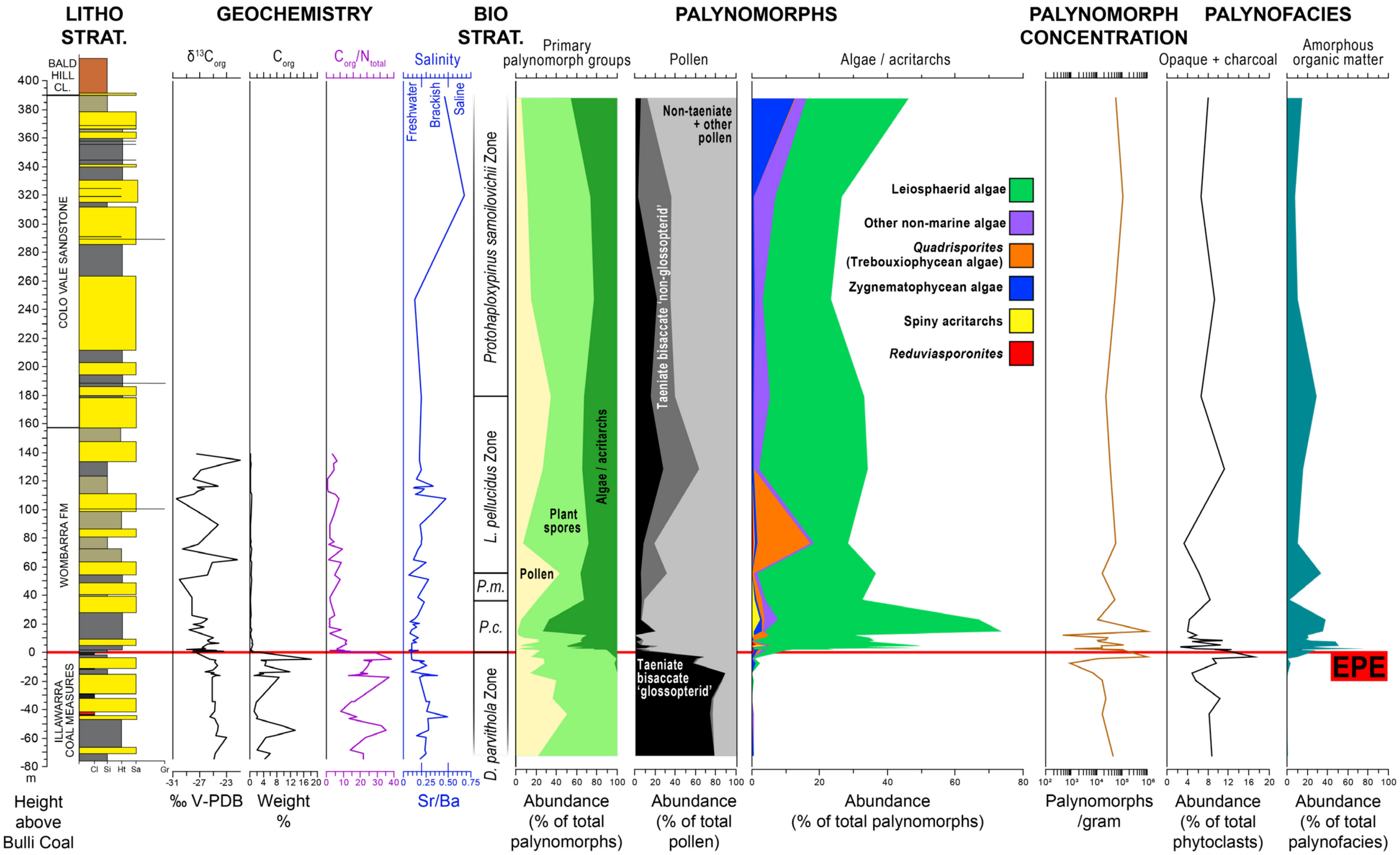

Supplementary Figure 1. **Lithostratigraphy (lithostrat.), geochemistry, spore-pollen biostratigraphy (palynostrat.), and palynological data of south-central Sydney Basin, Australia, as recorded from Bootleg-8**. Palynomorph concentration estimates calculated as per ref. [1]. See Supp. Data 12 for taxa in each palynomorph category. Algal affinities follow ref. [2]. For clarity, one sample (779.5 m, coal lithofacies) was excluded from the total organic carbon (C_{org}) graph because of its anomalously high value. Cl. = claystone, EPE = end-Permian extinction event (= upper horizon of the Bulli Coal), *D.* = *Dulhuntyispora*, *L.* = *Lunatisporites*, *P.c.* = *Playfordiaspora crenulata* Zone, *P.m.* = *Protohaploxypinus microcorpus* Zone, V-PDB = Vienna Peedee Belemnite.

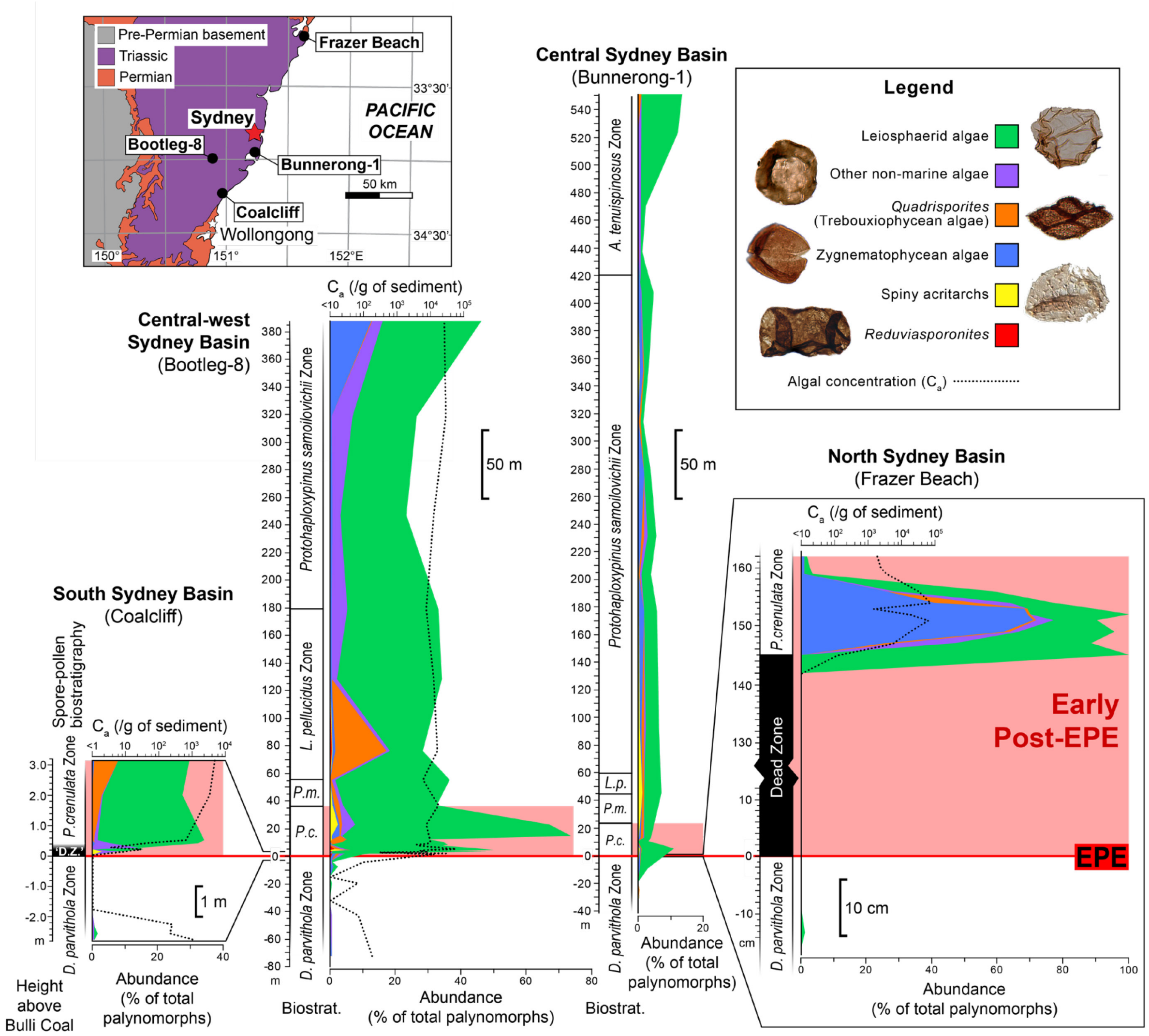

Supplementary Figure 2. **Algal abundances and concentrations across the end-Permian extinction interval (EPE) from the Sydney Basin, Australia.** The EPE is equivalent to the upper horizon of the Bulli Coal in each succession. Biostratigraphy sources: Bootleg-8 from this study, Coalcliff from ref. [3], Frazer Beach from ref. [4], Bunnerong-1 from ref. [5]. C_a = algal (+ acritarch) concentration per gram of dried sediment. Biozone abbreviations: *A.* = *Aratrisporites*, *D.* = *Dulhuntyispora*, *L.* = *Lunatisporites*, *P.c.* = *Playfordiaspora crenulata* Zone, *P.m.* = *Protohaploxypinus microcorpus* Zone. Concentration estimates in Supp. Data 5–7, algal abundances from Supp. Data 8–11, algal taxa for each category in Supp. Data 12. Algal affinities follow ref. [2].

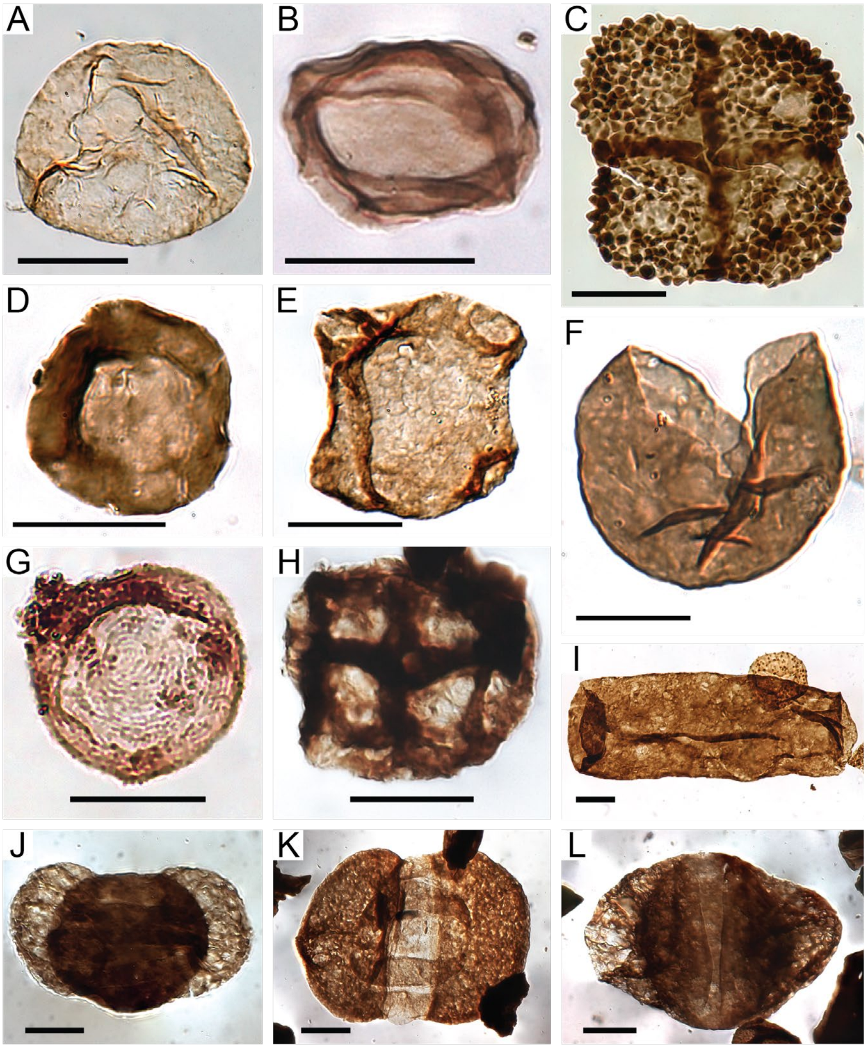

Supplementary Figure 3. **Pollen, freshwater algae and putative algae from the Lower Triassic continental deposits of the Sydney Basin, Australia**. All scales = 20 µm, algal affinities follow ref. [2], other affinities stated below. **A–H**, algal fossils; **A**, **B**, chlorophyte leiosphaerid alga, *Leiosphaeridia* sp.; **A**, Bootleg-8, 589.81 m, S029736/2 (U34); **B**, Bootleg-8, 766.18 m, S029752/2 (R38[4]); **C**, trebouxiophycean alga, *Quadrisporites horridus* Hennelly, 1958 [6] emend. Potonié & Lele, 1961 [7], Bootleg-8, 692.51 m, S029738/2 (W41[4]); **D**, other non-marine alga, *Pilasporites calculus* Balme & Hennelly, 1956 [8], Bootleg-8, 761.52 m, S029747/2 (T24); **E**, zygnematophycean alga, *Tetraporina* sp. cf. *T. protrusa* Brenner & Foster, 1994 [9], Bootleg-8, 381.16 m, S029733/2 (X37[3]); **F**, zygnematophycean alga, *Ovoidites scissus* (Balme & Hennelly, 1956 [8]) Zavattieri *et al*., 2020 [10], Bootleg-8, 692.51 m, S029738/2 (V43[2]); **G**, zygnematophycean alga, *Circulisporites* sp. A *sensu* Backhouse, 1991 [11], Frazer Beach, 152–153 cm, S200865/2; **H**, possible prasinophyte alga, *Cymatiosphaera* sp. cf. *C. gondwanensis* (Tiwari, 1965 [12]) Backhouse, 1991 [11], Bunnerong-1, 439.95 m, S014129/2 (Y43). **I**, putative algal (or fungal) fossil, *Reduviasporonites chalastus* (Foster, 1979 [13]) Elsik, 1999 [14], Bootleg-8, 692.51 m, S029738/2 (R33[4]). **J–L**, Pollen fossils; **J**, 'glossopterid-type' taeniate bisaccate pollen, *Protohaploxypinus samoilovichii* (Jansonius, 1962 [15]) Hart, 1964 [16], Bunnerong-1, 699.07 m, S014120/2 (J16[1]), this pollen is typical of glossopterids and some peltasperms [17]; **K**, 'non-glossopterid-type' taeniate bisaccate pollen, *Lunatisporites pellucidus* (Goubin, 1965 [18]) Helby in de Jersey, 1972 [19], Bunnerong-1, 362.00 m, S014132/2 (U14[3]), this pollen is typical of podocarpaceous [20] or voltzialean [21] conifers; **L**, non-taeniate bisaccate pollen, *Alisporites (Falcisporites) australis* de Jersey, 1962 [22], Bunnerong-1, 362.00 m, S014132/2 (N12[3]), this pollen form is typical of corystosperms and/or conifers [20, 23].

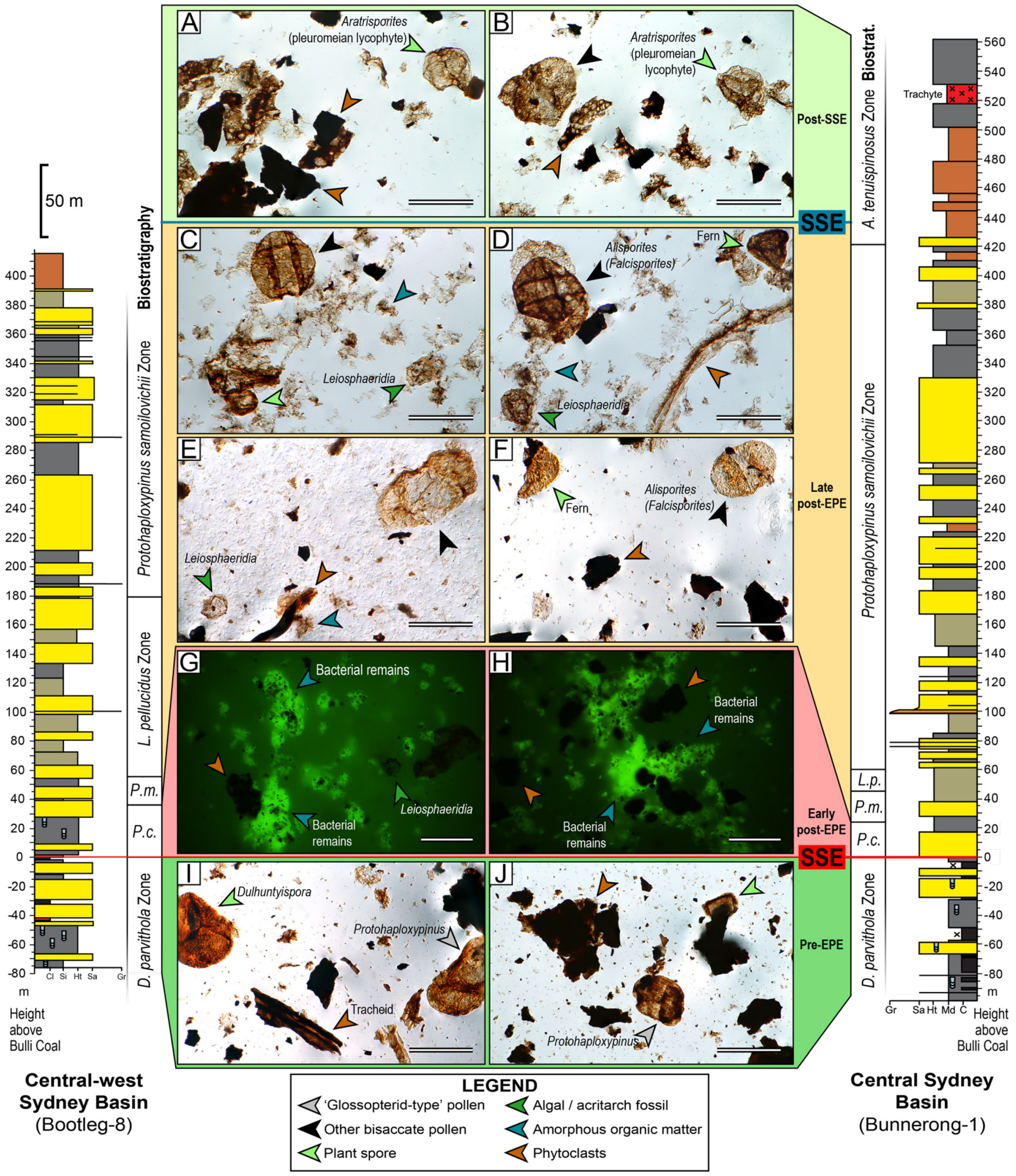

Supplementary Figure 4. **Representative palynofacies of the terrestrial ecosystem stages identified from the upper Permian to Lower Triassic of the Sydney Basin.** AOM typically represent primary bacterial accumulations, or the by-products of terrestrial matter degradation by microbes [24]; evidence for both of these AOM forms are present within the Sydney Basin. The high abundances of highly fluorescent AOM with granular texture in the early post-EPE interval indicate copious primary bacterial accumulation. All photomicrographs are of kerogen residues processed from siltstone lithofacies, all scales = 50 µm, spore/pollen affinities follow ref. [1], algal affinities follow ref. [2]. Bunnerong-1 stratigraphic log from ref. [5], and correlated to the biostratigraphic scheme of ref. [1]. EPE = end-Permian extinction event; SSE = Smithian-Spathian climatic event; palynostratigraphic zones: *A. = Aratrisporites*, *D. = Dulhuntyispora*, *L. = Lunatisporites*, *P.c. = Playfordiaspora crenulata* Zone, *P.m. = Protohaploxypinus microcorpus* Zone. **A**, **B**, optical, Bunnerong-1, 267.55 m, S014136/1; **C**, **D**, optical, Bunnerong-1, 493.95 m, S014129/1; **E**, optical, Bootleg-8, 713.34 m, S029739/1; **F**, optical, Lisarow-1, 446.8 m, S014000/1; **G**, blue light excitation fluorescence, Coalcliff outcrop, 197 to 205 cm, S029712/1; **H**, blue light excitation fluorescence, Coalcliff outcrop, 25 to 30 cm, S029708/1; **I**, optical, Lisarow-1, 649.52 m, S014019/1; **J**, optical, Bootleg-8, 788.37 m, S029760/1.

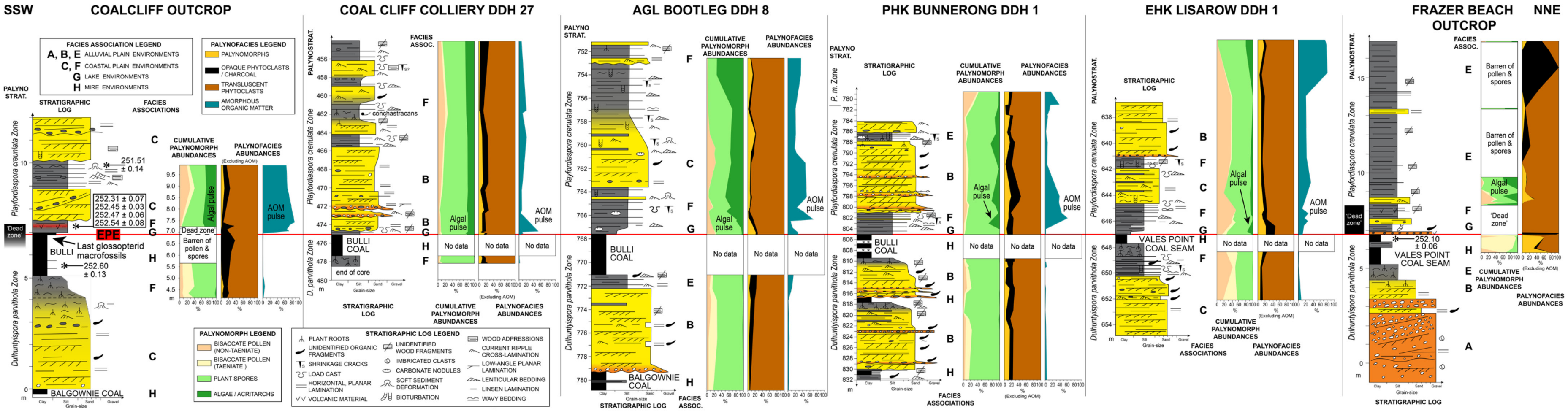

Supplementary Figure 5. **Stratigraphic and palynological records of the base of the end-Permian extinction interval (EPE) from selected successions across a SSW–NNE transect of the Sydney Basin** (see Fig. 1 for locations). Facies associations and environmental interpretations follow ref. [3]. Data sources: U-Pb radiogenic isotope age estimates (*, Ma) from refs [3, 5, 25]; stratigraphic logs from ref. [3] and this study; palynological data: Bootleg-8 from this study, CCO from this study and ref. [3], Coal Cliff Colliery DDH 27 (CCC-27) from ref. [1], Lisarow-1 from this study, FBO from this study and ref. [4], Bunnerong-1 from this study and ref. [1]. Figure updated from ref. [3].

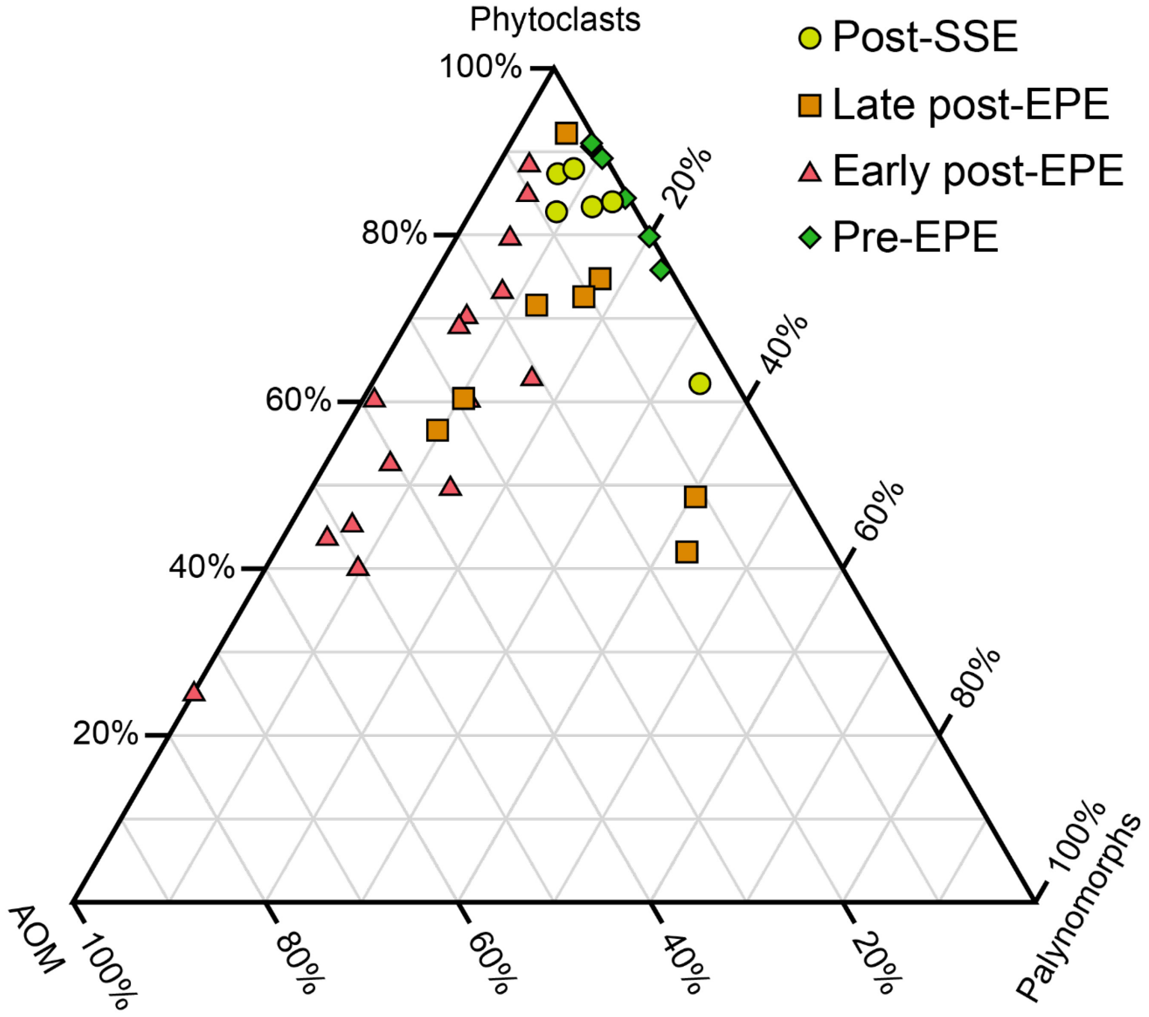

Supplementary Figure 6. **Ternary plot of the primary palynofacies categories**. Assemblages classified by ecological stage, EPE = end-Permian extinction event, SSE = Smithian-Spathian climatic event. Only those samples that meet the criteria for the ordination analysis have been included here.

References

1. Mays, C., *et al.* Refined Permian–Triassic floristic timeline reveals early collapse and delayed recovery of south polar terrestrial ecosystems. *GSA Bulletin* **132,** 1489–1513 (2020).
2. Mays, C., Vajda, V. & McLoughlin, S. Permian–Triassic non-marine algae of Gondwana—distributions, natural affinities and ecological implications. *Earth-Sci. Rev.* **212**, 103382 (2021).
3. Fielding, C. R., *et al.* Sedimentology of the continental end-Permian extinction event in the Sydney Basin, eastern Australia. *Sedimentology* **68,** 30–62 (2021).
4. Vajda, V., *et al.* End-Permian (252 Mya) deforestation, wildfires and flooding—an ancient biotic crisis with lessons for the present. *Earth Planet. Sci. Lett.* **529**, 115875 (2020).
5. Fielding, C. R., *et al.* Age and pattern of the southern high-latitude continental end-Permian extinction constrained by multiproxy analysis. *Nat. Comms* **10**, 385 (2019).
6. Hennelly, J. P. F. Spores and pollens from a Permian-Triassic transition, N.S.W. *Proc. Linn. Soc. NSW* **83,** 363–369 (1958).
7. Potonié, R. & Lele, K. M. Studies in the Talchir Flora of India, 1. Sporae dispersae from the Talchir Beds of South Rewa Gondwana Basin. *Palaeobotanist* **8,** 22–37 (1961).
8. Balme, B. E. & Hennelly, J. P. F. Monolete, monocolpate, and alete sporomorphs from Australian Permian sediments. *Aust. J. Bot.* **4,** 54–67 (1956).
9. Brenner, W. & Foster, C. B. Chlorophycean algae from the Triassic of Australia. *Rev. Palaeobot. Palynol.* **80,** 209–234 (1994).
10. Zavattieri, A. M., Gutiérrez, P. R. & Monti, M. Middle Triassic freshwater green algae and fungi of the Puesto Viejo Basin, central-western Argentina: Palaeoenvironmental implications. *Alcheringa* **44,** 430–459 (2020).
11. Backhouse, J. Permian palynostratigraphy of the Collie Basin, Western Australia. *Rev. Palaeobot. Palynol.* **67,** 237–314 (1991).
12. Tiwari, R. S. Miospore assemblage in some coals of Barakar Stage (Lower Gondwana) of India. *Palaeobotanist* **13,** 168–214 (1965).
13. Foster, C. B. Permian plant microfossils of the Blair Athol Coal Measures, Baralaba Coal Measures, and basal Rewan Formation of Queensland. *Geol. Surv. Qld. Pub.* **372,** 1–244 (1979).
14. Elsik, W. C. *Reduviasporonites* Wilson 1962: synonymy of the fungal organism involved in the Late Permian crisis. *Palynology* **23,** 37–41 (1999).
15. Jansonius, J. Palynology of Permian and Triassic sediments, Peace River area, western Canada. *Palaeontog. Abt. B* **110,** 35–98 (1962).
16. Hart, G. F. A review of the classification and distribution of the Permian miospore: Disaccate striatiti. *Compte Rendue 5th Congres International de Stratigraphie et de Geologie du Carbonifere* **3,** 1171–1199 (1964).
17. Balme, B. E. Fossil in situ spores and pollen grains: An annotated catalogue. *Rev. Palaeobot. Palynol.* **87,** 81–323 (1995).
18. Goubin, N. Description et répartition des principaux pollenites Permiens, Triasiques et Jurassiques des sondages du Bassin de Morondava (Madagascar). *Revue de L'Institut Français du Pétrole* **20,** 1415–1458 (1965).
19. de Jersey, N. J. Triassic miospores from the Esk Beds. *Geol. Surv. Qld. Pub.* **357,** 1–40 (1972).
20. Townrow, J. A. On *Rissikia* and *Mataia*, podocarpaceous conifers from the lower Mesozoic of southern lands. *Pap. Proc. Roy. Soc. Tas.* **101,** 103–138 (1967).
21. Townrow, J. A. On *Voltziopsis*, a southern conifer of Lower Triassic age. *Pap. Proc. Roy. Soc. Tas.* **101,** 173–188 (1967).
22. de Jersey, N. J. Triassic spores and pollen grains from the Ipswich Coalfield. *Geol. Surv. Qld. Pub.* **307,** 1–18 (1962).
23. Clement-Westerhof, J. A. *In situ* pollen from gymnospermous cones from the Upper Permian of the Italian Alps—A preliminary account. *Rev. Palaeobot. Palynol.* **17,** 63–73 (1974).

24. Pacton, M., Gorin, G. E. & Vasconcelos, C. Amorphous organic matter—Experimental data on formation and the role of microbes. *Rev. Palaeobot. Palynol.* **166,** 253–267 (2011).
25. Metcalfe, I., Crowley, J. L., Nicoll, R. S. & Schmitz, M. High-precision U-Pb CA-TIMS calibration of Middle Permian to Lower Triassic sequences, mass extinction and extreme climate-change in eastern Australian Gondwana. *Gondwana Res.* **28,** 61–81 (2015).
26. Traverse, A. *Paleopalynology*, Topics in Geobiology 28 (Springer, 2007).
27. Helby, R. J. *A Biostratigraphy of the Late Permian and Triassic of the Sydney Basin. Ph.D. thesis*, University of Sydney, Sydney, 513 pp. (1970). (unpublished).
28. de Jersey, N. J. Palynology of the Permian-Triassic transition in the western Bowen Basin. *Geol. Surv. Qld. Pub.* **374,** 1–39 (1979).
29. de Jersey, N. J. & Raine, J. I. Triassic and earliest Jurassic miospores from the Murihiku Supergroup, New Zealand. *New Zeal. Geol. Surv. Paleontol. Bull.* **62,** 1–164 (1990).
30. Balme, B. E. & Hennelly, J. P. F. Trilete sporomorphs from Australian Permian sediments. *Aust. J. Bot.* **4,** 240–260 (1956).
31. Potonié, R. Synopsis der Gattungen der Sporae dispersae. III. Teil: Nachträge Sporites, Fortsetzung Pollenites. Mit Generalregister zu Teil I–III. *Beihefte zum Geologischen Jahrbuch* **39,** 189 pp. (1960).
32. de Jersey, N. J. & Hamilton, M. Triassic spores and pollen grains from the Moolayember Formation. *Geol. Surv. Qld. Pub.* **336,** 1–61 (1967).
33. Balme, B. E. Palynology of Permian and Triassic strata in the Salt Range and Surghar Range, West Pakistan, in *Stratigraphic Boundary Problems—Permian and Triassic of West Pakistan*, Special Publications 4 (eds Kummel, B. & Teichert, C.), 305–453 (University of Kansas, 1970).
34. Wilson, L. R. Permian plant microfossils from the Flowerpot Formation, Greer County, Oklahoma. *Circulars of the Oklahoma Geology Survey* **49,** 1–50 (1962).
35. de Jersey, N. J. Early Triassic miospores from the Rewan Formation. *Geol. Surv. Qld. Pub.* **345,** 1–29 (1970).
