# Supplementary material for: Lethal microbial blooms delayed freshwater ecosystem recovery following the end-Permian extinction
Source: Nat Commun. 2021 Sep 17;12:5511. doi: 10.1038/s41467-021-25711-3 (PMC8448769; doi:10.1038/s41467-021-25711-3)
Supplement: Supplementary file 3 — Description of Additional Supplementary Files [file 41467_2021_25711_MOESM3_ESM.pdf]

Description of Additional Supplementary Files

Supplementary Data 1. Abundance table of palynofacies groups for the Sydney Basin well core succession AGL Bootleg DDH 8 (Bootleg-8), New South Wales, Australia. All percentages are calculated as a proportion of the respective palynofacies total, either category subtotal (n) or total palynofacies count (*N* = 500). Palyn = palynomorphs, phyto = phytoclasts, miospores = plant spores + pollen.

Supplementary Data 2. Abundance table of palynofacies groups for the Sydney Basin outcrop succession at Coalcliff (CCO), New South Wales, Australia. All percentages are calculated as a proportion of the respective palynofacies total, either category subtotal (n) or total palynofacies count (*N* = 500). Palyn = palynomorphs, phyto = phytoclasts, miospores = plant spores + pollen. * Comparisons between pre-EPE and post-EPE AOM abundances were not considered in this study, since the sample sizes were too small.

Supplementary Data 3. Abundance table of palynofacies groups for the Sydney Basin well core succession Elecom Hawkesbury Lisarow DDH 1 (Lisarow-1), New South Wales, Australia. All percentages are calculated as a proportion of the respective palynofacies total, either category subtotal (n) or total palynofacies count (*N* = 500). Palyn = palynomorphs, phyto = phytoclasts, miospores = plant spores + pollen.

Supplementary Data 4. Abundance table of palynofacies groups for the Sydney Basin outcrop succession at Frazer Beach (FBO), New South Wales, Australia. All percentages are calculated as a proportion of the respective palynofacies total, either category subtotal (n) or total palynofacies count (*N*). Palyn = palynomorphs, phyto = phytoclasts, miospores = plant spores + pollen.

Supplementary Data 5. Abundance table of palynomorph groups for AGL Bootleg DDH 8 (Bootleg-8), New South Wales, Australia. All percentages are calculated as a proportion of the respective total palynomorph sample count (*N*), and estimated from oxidised and sieved palynomorph sample counts.

Supplementary Data 6. Abundance table of palynomorph groups for the outcrop succession at Coalcliff (CCO), New South Wales, Australia. All percentages are calculated as a proportion of the respective total palynomorph sample count (*N*), and estimated from oxidised and sieved palynomorph sample counts.

Supplementary Data 7. Abundance table of palynomorph groups for the outcrop succession at Frazer Beach (FBO), New South Wales, Australia. All percentages are calculated as a proportion of the respective total palynomorph sample count (*N*), and estimated from oxidised and sieved palynomorph sample counts.

Supplementary Data 8. Algal fossil group abundances for AGL Bootleg DDH 8 (Bootleg-8), New South Wales, Australia. All percentages are calculated as a proportion of the respective total palynomorph sample count (*N*), and estimated from oxidised and sieved palynomorph sample counts.

Supplementary Data 9. Algal fossil group abundances for the outcrop succession at Coalcliff (CCO), New South Wales, Australia. All percentages are calculated as a proportion of the respective total palynomorph sample count (*N*), and estimated from oxidised and sieved palynomorph sample counts.

Supplementary Data 10. Algal fossil group abundances for Pacific Power Hawkesbury DDH 1 (Bunnerong-1), New South Wales, Australia. All percentages are calculated as a proportion of the

respective total palynomorph sample count (*N*), and estimated from oxidised and sieved palynomorph sample counts.

Supplementary Data 11. Algal fossil group abundances for the outcrop succession at Frazer Beach (FBO), New South Wales, Australia. All percentages are calculated as a proportion of the respective total palynomorph sample count (*N*), and estimated from oxidised and sieved palynomorph sample counts.

Supplementary Data 12. Palynomorph taxon groups employed in this paper (modified from ref. [1]). Taxa in bold have been employed herein for regional palynostratigraphic correlation. Spore and pollen morphological categories follow ref. [2], algal and acritarch categories and affinities follow ref. [3]. "Spp." includes only other species not already listed.

Supplementary Data 13. Geochemical data for Pacific Power Hawkesbury DDH 1 (Bunnerong-1), New South Wales, Australia.

Supplementary Data 14. Geochemical data for the outcrop succession at Frazer Beach (FBO), New South Wales, Australia.

Supplementary Data 15. Geochemical data for the AGL Bootleg DDH 8 (Bootleg-8), New South Wales, Australia.

Supplementary Data 16. Geochemical data for the AGL Bootleg DDH 8 (Bootleg-8), New South Wales, Australia. Linear regression analyses between geochemical proxies and palynofacies or palynomorph abundances are provided; statistical analyses only included samples where coeval geochemical and palynological data were collected.
