# Supplementary material for: Lethal microbial blooms delayed freshwater ecosystem recovery following the end-Permian extinction
Source: Nat Commun. 2021 Sep 17;12:5511. doi: 10.1038/s41467-021-25711-3 (PMC8448769; doi:10.1038/s41467-021-25711-3)
Supplement: Supplementary file 20 — Reporting Summary [file 41467_2021_25711_MOESM20_ESM.pdf]

nature research

Corresponding author(s): Chris Mays

Last updated by author(s): Aug 4, 2021

Reporting Summary

Nature Research wishes to improve the reproducibility of the work that we publish. This form provides structure for consistency and transparency in reporting. For further information on Nature Research policies, see our Editorial Policies and the Editorial Policy Checklist.

Statistics

For all statistical analyses, confirm that the following items are present in the figure legend, table legend, main text, or Methods section.

n/a	Confirmed	
x		The exact sample size (n) for each experimental group/condition, given as a discrete number and unit of measurement
	x	A statement on whether measurements were taken from distinct samples or whether the same sample was measured repeatedly
	x	The statistical test(s) used AND whether they are one- or two-sided<br>*Only common tests should be described solely by name; describe more complex techniques in the Methods section.*
x		A description of all covariates tested
	x	A description of any assumptions or corrections, such as tests of normality and adjustment for multiple comparisons
	x	A full description of the statistical parameters including central tendency (e.g. means) or other basic estimates (e.g. regression coefficient) AND variation (e.g. standard deviation) or associated estimates of uncertainty (e.g. confidence intervals)
	x	For null hypothesis testing, the test statistic (e.g. F, t, r) with confidence intervals, effect sizes, degrees of freedom and P value noted<br>*Give P values as exact values whenever suitable.*
x		For Bayesian analysis, information on the choice of priors and Markov chain Monte Carlo settings
x		For hierarchical and complex designs, identification of the appropriate level for tests and full reporting of outcomes
	x	Estimates of effect sizes (e.g. Cohen's d, Pearson's r), indicating how they were calculated

Our web collection on statistics for biologists contains articles on many of the points above.

Software and code

Policy information about availability of computer code

Data collection	No software was used.
Data analysis	PAST - PAlaeontological STatistics 4.03 (open access).

For manuscripts utilizing custom algorithms or software that are central to the research but not yet described in published literature, software must be made available to editors and reviewers. We strongly encourage code deposition in a community repository (e.g. GitHub). See the Nature Research guidelines for submitting code & software for further information.

Data

Policy information about availability of data

All manuscripts must include a data availability statement. This statement should provide the following information, where applicable:

- Accession codes, unique identifiers, or web links for publicly available datasets
- A list of figures that have associated raw data
- A description of any restrictions on data availability

The authors declare that all data supporting the findings of this study are available within the paper and its supplementary information files. Palynological slides used in this study are held in the collections of the Swedish Museum of Natural History (SMNH), Stockholm, under the following registration codes: S014000–S014027 (Lisarow-1), S014101–S014148 (Bunnerong-1), S029733–S029763 (Bootleg-8), S029700–S029713 (Coalcliff outcrop), S200841–S200901 (Frazer Beach outcrop). Core samples and plant fossil records are held in the W.B. Clarke Geoscience Centre drillcore library, Londonderry, New South Wales, Australia.

Field-specific reporting

Please select the one below that is the best fit for your research. If you are not sure, read the appropriate sections before making your selection.

- [ ] Life sciences
- [ ] Behavioural & social sciences
- [x] Ecological, evolutionary & environmental sciences

For a reference copy of the document with all sections, see nature.com/documents/nr-reporting-summary-flat.pdf

Ecological, evolutionary & environmental sciences study design

All studies must disclose on these points even when the disclosure is negative.

Study description	Microfossil, sediment and geochemical data sets were collected from a series of stratigraphic successions spanning the target geological interval (the upper Permian to Lower Triassic). Sedimentary data were collected with continuous lithological logging, and facies associations based on a previously published framework from the target region. These sedimentary data were collected first to allow targeting of the appropriate lithofacies for microfossils and geochemistry. Fossil and geochemical data were then collected from the same samples to facilitate valid comparisons, but these were processed and analysed independently and concurrently. Details of sample sizes and treatments are outlined in the Methods.
Research sample	Organic micropalaeontology targets: plant spores, pollen, leaves, algae, bacteria from macerated sedimentary samples;<br>Organic micropalaeontology rationale: 1, each sample in a given stratigraphic succession comes from a distinct time of deposition; 2, each of the fossil assemblages is indicative of the suite of organically preserved primary producers (algae, bacteria, plants) for each interval; and 3, a vertical series of these fossils represents the changing abundances of these organisms over time.<br>Sedimentary geology targets: lithofacies, specifically sedimentary textures, sedimentary structures and trace fossils.<br>Sedimentary geology rationale: the facies reflect the local depositional environments and the assemblage of animal trace makers; their vertical succession represent changes in these conditions and assemblages.<br>Geochemistry targets: isotopic and elemental concentrations derived from sedimentary samples.<br>Geochemistry rationale: by collecting these data from the same samples as the organic micropalaeontology and sedimentary geology (above), they provide additional, independent proxies for the changes in primary producers and local conditions.
Sampling strategy	Sedimentary rock successions for this study came from both outcrops and bore-cores. Bore-cores are held in a publicly accessible repository, the Londonderry Drillcore Library (NSW Resources and Geoscience, Australia). All sedimentary geology data were collected directly from observations of these successions. For microfossil and geochemical data, rock samples were collected from target intervals using manual extract tools (e.g., hammers, chisels, rock saws).<br>Where possible, microfossil counts consisted of sample sizes that were greater than standard palynological population counts for palaeoecological applications (as demonstrated by previously published studies; see Traverse, 2007). Samples that failed to meet predetermined minimum count sizes were indicated, and excluded from statistical analyses.
Data collection	For specifics of sample collection, see 'sampling strategy' above. All fossil and geochemical samples underwent processing by the same labs to minimise variability caused by differential preparation. Geochemical data were compared to a series of previously published international and in-house standards (see Methods).
Timing and spatial scale	Samples were collected and processed in two subsequent phases. The first sampling phase (May 2017) consisted of wide-interval spacing (vertical axis) of the entire stratigraphic succession from the bore-cores. The purpose of this was to provide a broad overview of the trends for the entire stratigraphic succession. The second phase (one field season: Nov-Dec 2018) included targeted outcrop sampling for high-resolution data across the end-Permian extinction interval. This phased sampling and analysis approach was conducted to reveal the data trends from the stratigraphic intervals of greatest interest.<br>To test for spatial variability, the data were collected from five stratigraphic successions in a 130-kilometer-long, approximately NNE-SSW transverse axis across the Sydney Basin.
Data exclusions	Organic micropalaeontology: All collected data have been included in the data set. However, some samples were excluded from the statistical analysis and illustrations based on the following pre-established criteria: 1, the fossil assemblage was derived from samples of sandstone or coarser sediment grain-size (to minimise mechanical fossil reworking and excess post-burial alteration via oxidation); and/or 2, the assemblages failed to meet the sample count minimum (see above).
Reproducibility	Microfossil data trends were reproduced by two different palynologists (CM and VV) from five successions across the target region to ensure the validity of methods and results. All fossil specimens are housed within the publicly accessible collections of the Swedish Museum of Natural History. Sedimentary and geochemical data may be replicated by sample analysis of the precise stratigraphic heights indicated herein. Geochemical analyses were conducted by comparing the results to international standard samples. To ensure reproducibility of the geochemical results, analyses were monitored through replicate analyses of standards and samples; confidence intervals for these analyses are provided (see Methods for details).
Randomization	Randomization was not applicable. The samples analysed consisted of a time series, and all samples were included in the comparisons.
Blinding	Blinding during microfossil and geochemical data collection was achieved by conducting independent and concurrent analyses.

Did the study involve field work? [x] Yes [ ] No

Field work, collection and transport

Field conditions	Mild weather on coastal outcrops; no rainfall, temperatures: 15–25°C.
Location	Coalcliff outcrop (34° 15' 18.9"S, 150° 58' 22.2"E), c. 10–15 m above sea level; Frazer Beach outcrop (33° 11' 37.2"S, 151° 37' 22.3"E), <12 m above sea level.
Access & import/export	All field samples were collected by removing >20cm of surface rock to eliminate modern contaminants. Resulting samples were clean of soil or living biological material. Sampling had minimal environmental impact, and did not need approval from the NSW Government prior to collection (see below: "Reporting for specific materials, systems and methods").
Disturbance	All target areas were on steep cliff surfaces (>60 degrees) with minimal signs of living biological activity. Small areas were targeted for each outcrop sample (<10 x 10 cm).

Reporting for specific materials, systems and methods

We require information from authors about some types of materials, experimental systems and methods used in many studies. Here, indicate whether each material, system or method listed is relevant to your study. If you are not sure if a list item applies to your research, read the appropriate section before selecting a response.

Materials & experimental systems

n/a	Involved in the study	
☒	☐	Antibodies
☒	☐	Eukaryotic cell lines
☐	☒	Palaeontology and archaeology
☒	☐	Animals and other organisms
☒	☐	Human research participants
☒	☐	Clinical data
☒	☐	Dual use research of concern

Methods

n/a	Involved in the study	
☒	☐	ChIP-seq
☒	☐	Flow cytometry
☒	☐	MRI-based neuroimaging

Palaeontology and Archaeology

Specimen provenance	Palynological samples were prepared from mudrock specimens of drillcores or outcrops.<br>Drillcores and leaf specimens are housed at the W.B. Clarke Geoscience Centre drillcore library in Londonderry, New South Wales (NSW), Australia.<br>Other samples were collected from coastal outcrops within national parks of NSW. These areas are under the jurisdiction of the NSW Government. Samples collected with minimal environmental impact from NSW Government land do not need approval prior to collection, as indicated by the State Environmental Planning Policy (Mining, Petroleum Production and Extractive Industries; Mining SEPP, 2007). Because they were sampled 'using hand-held equipment' (Mining SEPP, 2007), this process had minimal environmental impact.
Specimen deposition	Palynological slides are lodged at the Swedish Museum of Natural History, Stockholm.
Dating methods	No new absolute age constraints provided. New relative age constraints (non-numerical biozones) for strata based on spore-pollen biostratigraphic correlations to the regional chronostratigraphic scheme. Citations to this scheme are provided where relevant.

☐ Tick this box to confirm that the raw and calibrated dates are available in the paper or in Supplementary Information.

Ethics oversight	No ethics approval was required. All samples were obtained following NSW Government regulations, and the provenance and repositories of these samples are outlined in the manuscript.

Note that full information on the approval of the study protocol must also be provided in the manuscript.
